# Inclusion of modified nano-magnesium hydroxide as an adjuvant flame retardant in the development of PLA/hydroxyapatite nanocomposites

**DOI:** 10.1016/j.heliyon.2024.e39529

**Published:** 2024-10-18

**Authors:** Mohsen Hajibeygi, Fatemeh Darvishi

**Affiliations:** Department of Organic and Polymer Chemistry, Faculty of Chemistry, Kharazmi University, 15719-14911, Tehran, Iran

**Keywords:** Polylactic acid, Hydroxyapatite, Mg(OH)_2_ nanoparticle, Thermal stability, Flame-retardant

## Abstract

For the preparation of thermal stable and flame-retardant polylactic acid (PLA) nanocomposites two new modified nano-structures including imide-carboxylated chitosan modified Mg(OH)_2_ (ICMH) and imide-silane functionalized hydroxyapatite nanoparticles (ISHA) were prepared. The structure, morphology, and properties of ICMH and ISHA were investigated using Fourier transform infrared spectroscopy (FTIR), X-ray diffraction (XRD), Field-emission scanning electron microscopy (FE-SEM), and thermogravimetric analysis (TGA). The PLA nanocomposites were prepared via solution casting method. The results of Transmission electron microscopy (TEM) and SEM indicated a homogeneous structure for the PLA nanocomposites. The TGA results revealed that the presence of hydroxyapatite nanoparticles (HA) led to increase the thermal resistance of the PLA nanocomposites containing Mg(OH)_2_ (MDH). Compared to pure PLA, the T_max_ value of the PLA sample containing 3 wt% of each filler (the PMH6 sample) enhanced from 364 °C to 372 °C. The results from the microscale combustion calorimeter (MCC) test illustrated that the key parameter values of the PLA nanocomposites were reduced, as compared to the pure PLA. The peak heat release rate (pHRR) and heat release capacity (HRC) values of PMH6 decreased 27 % and 35 %, respectively, as compared to the pure PLA. Also, flammability of PMH6 was reduced, as compared to the pure PLA, with UL-94 V-1 rating and LOI value 26.8 %. Furthermore, the tensile strength of the mentioned sample increased from 36.04 MPa to 38.58 MPa.

## Highlights

• Preparation of imide-carboxylated chitosan modified Mg(OH)_2_ (ICMH).
• Preparation of PLA/modified hydroxyapatite nanocomposites.
• Study the impact of ICMH on the properties of PLA/HA nanocomposite.
• To improve heat resistance and flammability both nanofillers were necessary.
• Study on mechanical properties of resulted PLA nanocomposites.

## Graphical abstract

**Figure:**
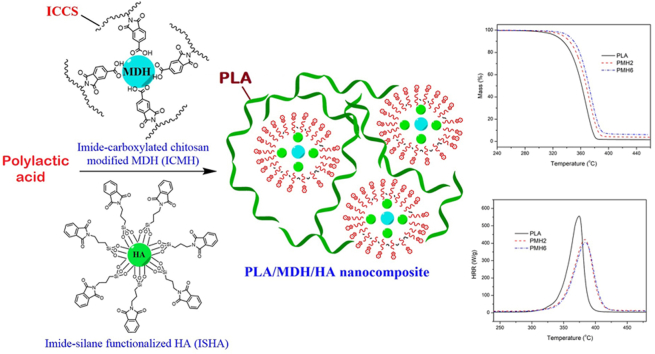

## 1 Introduction

The widespread use of materials derived from petroleum leads to significant environmental pollution due to the over-reliance on and depletion of oil resources [1]. Therefore, attention to the creation and increasing the application of biodegradable and renewable polymers is crucial to replace conventional polymers derived from petroleum [2]. Polylactic acid (PLA), as a biodegradable and renewable polymer, can be produced from all-natural sources such as corn, sugar, and potatoes [3,4]. Due to having exclusive properties including biodegradability, composability, high transparency, thermal stability, and excellent mechanical strength [5,6], PLA has been utilized in various applications including electronic appliances [7], food containers [8], packaging films [9], bone fixation material [10], and as filament for 3D printers [11]. PLA is naturally flammable with a LOI of 18–20 percent due to its underlying chemical and molecular structure, which has limited its use in certain thriving high-tech industries such as packaging, electrical devices, and textiles, etc. [12]. To reduce the flammability of PLA, a significant quantity of flame retardant (FR) has been employed to enhance its fire resistance. Nevertheless, this led to a decline in the thermal resistance and mechanical strength of PLA due to the excessive FR loadings. Therefore, it is imperative to select a simple and economical method to preserve the equilibrium between flame retardancy and mechanical characteristics of biodegradable polymer composites [13,14].

Over the past ten years, PLA-based nanocomposites have demonstrated enhanced thermal and flame retardancy characteristics, which have been further enhanced by the use of various inorganic nanomaterials, including hydroxyapatite nanoparticles (HA), tricalcium phosphate, bioactive glass, and others [[15], [16], [17]]. To achieve desirable properties such as thermal stability and flame retardancy, hydroxyapatite (HA) has reportedly been incorporated into several polymers, including PLA [18], poly(methyl methacrylate) (PMMA) [19], polycarbonate (PC) [20], polypropylene (PP) [21], and epoxy resin [22]. Because of the high biocompatibility in PLA/HA composites, HA is mostly used in medical applications [23], but mixing it with other useful organic and inorganic materials, such as graphene oxide (GO) and magnesium oxide (MgO), can obtain effective additives for different polymers such as PLA [24,25]. Based on the characteristics of MgO and Mg(OH)₂ (MDH), the flame retardant effect observed in MDH is not present in MgO [26], therefore, MDH can be a suitable option for improving the combustion resistance of PLA.

MDH nanoparticles, employed as an additive devoid of smoke and toxins, have widespread application in halogen-free flame-retardant polymers [27,28]. MDH endothermic decomposes into water and MgO at approximately 350 °C, a process that absorbs a significant amount of heat and lowers the material's temperature [29]. Moreover, the resulting magnesium oxide is a gel-like substance that continues to hydrate during the re-curing process, thereby enhancing strength recovery [30]. Therefore, it has been employed as a flame-retardant additive due to its beneficial properties, but to the knowledge of the authors of this research, it has not been utilized singly for improvement the thermal stability of PLA. The non-use of MDH may be due to its decomposition and water loss at the same time as the thermal degradation temperature of PLA, which leads to a decrease of the PLA thermal resistance [31]. Although MDH has harmed the thermal resistance of PLA, it has led to an increase in its flame retardancy. The decrease in thermal resistance can be compensated for by blending MDH with other effective materials such as HA and surface modification of inorganic nanomaterials with organic compounds containing suitable functional groups.

Controlling the nano-structure of nanocomposites presents challenges, specifically the level of filler dispersion, which can limit the properties and application of polymer nanocomposites. Coupling agents or surfactants were usually employed for surface treatment and to enhance the compatibility between nanoparticles and polymer matrix [32,33]. Various renewable and biodegradable materials, such as sodium alginate, β-cyclodextrin, sodium lignosulfonate, and chitosan, have been employed for the surface treatment of nano-structured materials based on their applications [[34], [35], [36], [37]]. Chitosan (CS), as a D-glucosamine polysaccharide, is derived from chitin and has been known for its various useful properties such as antibacterial, mucoadhesive, antifungal, and hemostatic properties [37]. CS has consequential applications in pharmaceutical, agricultural, material science, environmental, food industries, coating applications, membranes, hydrogels, nanofibers/scaffold, and nanocomposites [38]. Several modifications and derivatization of CS have been reported for different applications such as surface modification of nanomaterials for the preparation of polymer nanocomposites [[39], [40], [41]].

This project utilizes modified MDH and HA to create PLA nanocomposites, assessing their thermal, flammability, and mechanical properties. The structure of chitosan was imide functionalized and used for the surface modification of MDH. The modified MDH was used as an adjuvant flame-retardant for preparation of PLA/MDH/HA nanocomposites. HA nanoparticles was treated with aminopropyl silane and imide functionalization. It was expected that HA, as an effective additive, could also compensate for the reduction in thermal resistance caused by the presence of MDH.

## 2 Experimental

### 2.1 Materials

Trimellitic anhydride, phthalic anhydride, aminopropyl triethoxy silane, calcium nitrate‐tetrahydrate, magnesium nitrate-hexahydrate, dimethyl formamide (DMF), diammonium hydrogen phosphate, ammonia solution 25 % were purchased from Merk Company. Chitosan with medium molecular weight (MW: 190–310 kDa, degree of deacetylation: 75–85 %) was provided from Sigma-Aldrich. PLA (type 4042D) was obtained from American Nature works.

### 2.2 Measurements

FTIR spectra was recorded by PerkinElmer RXI spectrometer. XRD patterns recorded with device STOE STIDY MP with specification Cu, KA1: 1.5405 A°. Tensile test was done with the SANTAM STM-50 machine and the specifications are as follows: at 25 °C, speed of 5 mm min^−1^ and the dimensions of the PLA and PLA nanocomposite films used in this test were 20 mm × 60 mm and their thickness were between 0.7 and 0.9 mm (ASTM D882). The test was repeated for each sample three times. TGA was performed with a TGAQ5000 (TA instrument) and the samples were examined in the following conditions: in N_2_ atmosphere at a heating rate 10 °C.min^−1^. Differential scanning calorimetry (DSC) analysis was performed with a Q1000 (TA instrument) and the samples were examined in the following conditions: in N_2_ atmosphere and heating rate 10 °C.min^−1^. The morphology of the multifunctional nanohybrid materials was investigated by FE-SEM (Mira3TESCAN-XMU) and the morphology of the PLA nanocomposite films was investigated with SEM NEON40 SEM. A TEM with a 200 kV Schottky HR field emission was used for investigation of the structure of the PLA sample. In order to study the flame retardancy, 5 mg of the sample was examined with heating rate of 1 °C.s^−1^ by MCC (MCC-1, FTT). The test was repeated for each sample three times. The Limiting Oxygen Index (LOI) measurements were performed for the samples with dimensions: 100 mm long, 10 mm wide, and 1.0–1.5 mm thickness using a Candle type flammability tester model D made by Toyo Seiki Seisaku-SHO Ltd., Japan (ASTM D2863). The vertical UL-94 test was done for the samples with dimensions: 120 mm long, 10 mm wide, and 0.7–0.9 mm thickness according to the ASTM D4804 standard.

### 2.3 Preparation of imide-carboxylated chitosan modified Mg(OH)_2_ (ICMH)

#### 2.3.1 Preparation of imide-carboxylated chitosan (ICCS)

In a 50-mL flask, 0.3 g CS and 0.3 g trimellitic anhydride were dissolved in 28 mL of DMF. The solution was kept at room temperature overnight and then heated at 120 °C for 5 h. The resulting imide-carboxylated chitosan (ICCS) solution was cooled to room temperature. Meanwhile, MDH that was prepared by the co-precipitation method [42], was surface-modified with the ICCS solution as follows.

#### 2.3.2 Preparation of ICMH

In a 100-mL beaker, 0.35 g MDH and 5 mL DMF were added and stirred at 25 °C for 1 h. The mixture was then irradiated with ultrasonic waves for 10 min before being added to the ICCS solution. The MDH suspension was added to the ICCS solution. The resulting suspension was stirred at 40 °C for 24 h and irradiated with ultrasonic waves several times. The product was then filtered and dried completely in a vacuum oven at 50 °C for 24 h.

### 2.4 Preparation of imide-silane functionalized hydroxyapatite nanoparticles (ISHA)

#### 2.4.1 Preparation of amino-silane hydroxyapatite nanoparticles (ASHA)

HA was prepared by the co-precipitation method according to previous literature [43]. In a 50-mL flask, 0.5 g HA was mixed with 5 mL dry toluene and stirred for 1 h, followed by 10 min of ultrasonic treatment. Then, 0.6 mL aminopropyl triethoxysilane was added, and the mixture was heated at 80 °C for 3 h. The temperature of the resulting ASHA suspension was reduced to 25 °C, and after filtration, it was washed several times with dry toluene.

#### 2.4.2 Preparation of ISHA

In a 50-mL flask, 0.25 g ASHA and a solution containing 0.25 g phthalic anhydride in 4 mL DMF were added. The resulting mixture was stirred at 25 °C for 24 h and irradiated with ultrasonic waves several times. It was then heated at 80 °C for 5 h. After cooling to 25 °C, the mixture was filtered and washed with a 1:1 solution of DMF and ethanol.

### 2.5 Preparation of PLA/MDH/HA nanocomposites (PMHN)

In this study, nanocomposites of PLA and surface-modified nanostructures of HA and MDH were prepared via the solution casting method. The preparation method for PLA nanocomposite containing one wt% of each filler is described as a sample procedure for the fabrication of these nanocomposites.

In a 100-mL flask, 0.015 g ICMH and 0.015 g ISHA were combined with 8 mL of DMF and stirred at 25 °C for 24 h. During this time, it was subjected to ultrasonic waves several times. Then, 1.47 g of PLA was dissolved in 25 mL DMF at 100 °C, and after cooling to room temperature, it was added to the mixture of ICMH and ISHA. The resulting mixture was stirred at 25 °C for 24 h and subjected to ultrasonic waves several times. Finally, the nanocomposite mixture was poured into a Petri dish, and to evaporate the solvent and process the PMH2 film, it was placed in a vacuum oven at 75 °C. The remaining PLA nanocomposite films were prepared using the same method. The wt% and type of the fillers are presented in Table 1.

**Table 1:** Formulation of the PLA nanocomposites.

Sample	PLA (wt%)	ICMH (wt%)	ISHA (wt%)
PLA	100	0	0
PM2	98	2	0
PM6	94	6	0
PH2	98	0	2
PH6	94	0	6
PMH2	98	1	1
PMH6	94	3	3

PLA: polylactic acid, ICMH: Imide-carboxylated chitosan modified Mg(OH)_2_, ISHA: Imide-silane functionalized hydroxyapatite nanoparticles.

## 3 Results and discussion

### 3.1 Characterization of ICMH and ISHA

**Scheme 1:**
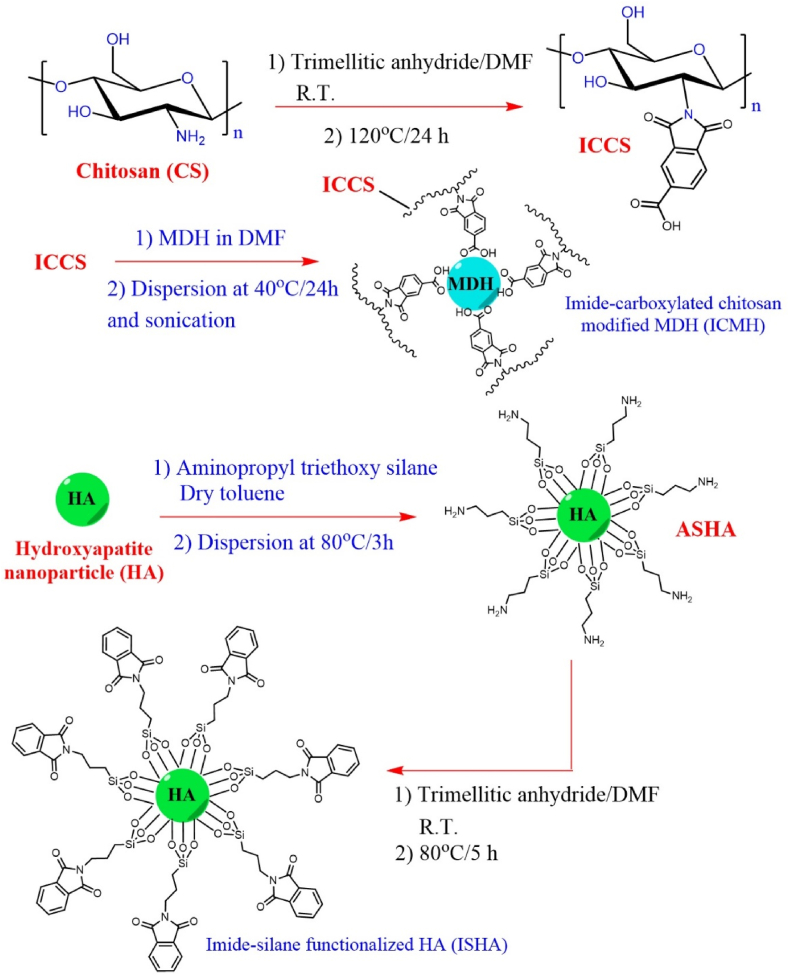
Preparation route of surface modified MDH (top) and HA (down).

At first, MDH was surface-modified with imide functionalized chitosan. The presence of an imide heterocyclic structure as a pendant group on the CS chains can increase the thermal stability of CS. To achieve the imide-functionalized chitosan, CS was reacted with trimellitic anhydride acid in DMF as the solvent. The preparation route of ICMH is shown in the top of Scheme 1. On the other hand, the surface of HA was modified by imide functionalized silane. For the amine functionalization of HA, HA was reacted with amino propyl silane and then with phthalic anhydride. In this process, the polarity of HA can increase, and the imide heterocyclic structure may lead to increased thermal stability of the resulting PLA nanocomposite. The preparation route of ISHA is also shown in the bottom of Scheme 1. The structures and morphologies of ICMH and ISHA were examined using different analyses including FTIR, XRD, FE-SEM, and TGA.

**Fig. 1:**
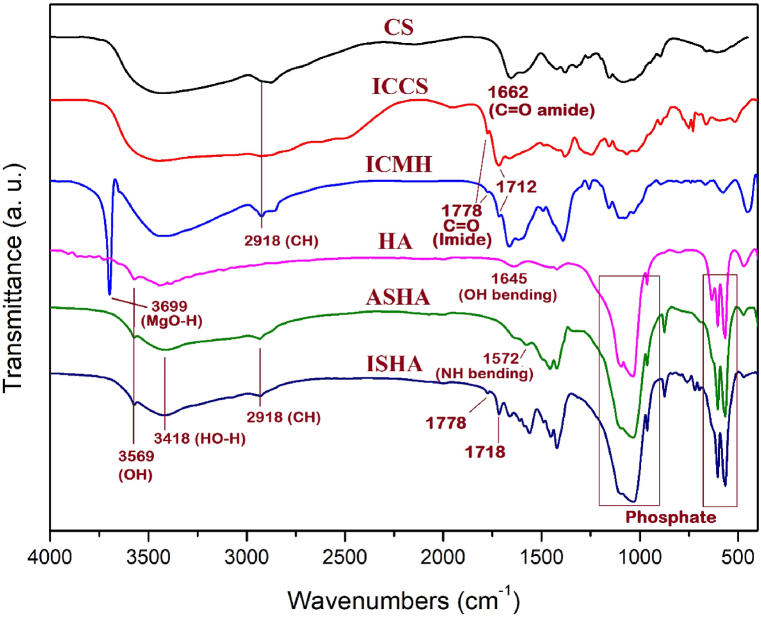
FTIR spectra of CS, ICCS, ICMH, HA, ASHA, and ISHA.

Infrared spectroscopy was utilized to analyze the molecular structure and confirm the presence of specific bonds. The FTIR spectra of CS, ICCS, ICMH, HA, ASHA, and ISHA are shown in Fig. 1. In the CS spectrum, the band at 3400 cm^−1^ revealed the O-H stretching vibration, typical of hydroxyl groups. A sharp absorption band observed at 1662 cm^−1^ corresponded to the C=O stretching vibration, suggesting the presence of a carbonyl amide group in the CS structure. After the imide functionalization of CS, two absorption bands related to symmetric and antisymmetric carbonyl in the imide structure of ICCS appeared at 1778 cm^−1^ and 1712 cm^−1^, respectively. In the spectrum of ICMH, the intense absorption band at 3699 cm^−1^ is indicative of MgO-H stretching vibrations, suggesting the presence of the MDH structure. The absorption bands at 2918 cm^−1^ and 2889 cm^−1^ can be attributed to aliphatic CH stretching modes, related to the CS structure. In the FTIR spectrum of ISHA, a weak absorption band at 3569 cm^−1^ is related to the OH groups in HA structure. The absorption band at 3414 cm^−1^ revealed the O-H stretching vibration for the hydroxyl groups in the adsorbed water molecules. The phosphate ions stretching modes are appeared at 563 cm^−1^, 600 cm^−1^, and 1028 cm^−1^. The stretching mode for carbonyl imide groups are observed at 1778 cm^−1^ and 1718 cm^−1^. The stretching vibration related to O-Si-O typically observed at 1130 cm^−1^, which overlapped with phosphate absorption bands. Overall, the FTIR spectra provided compelling evidence for functional groups consistent with the proposed molecular structure.

**Fig. 2:**
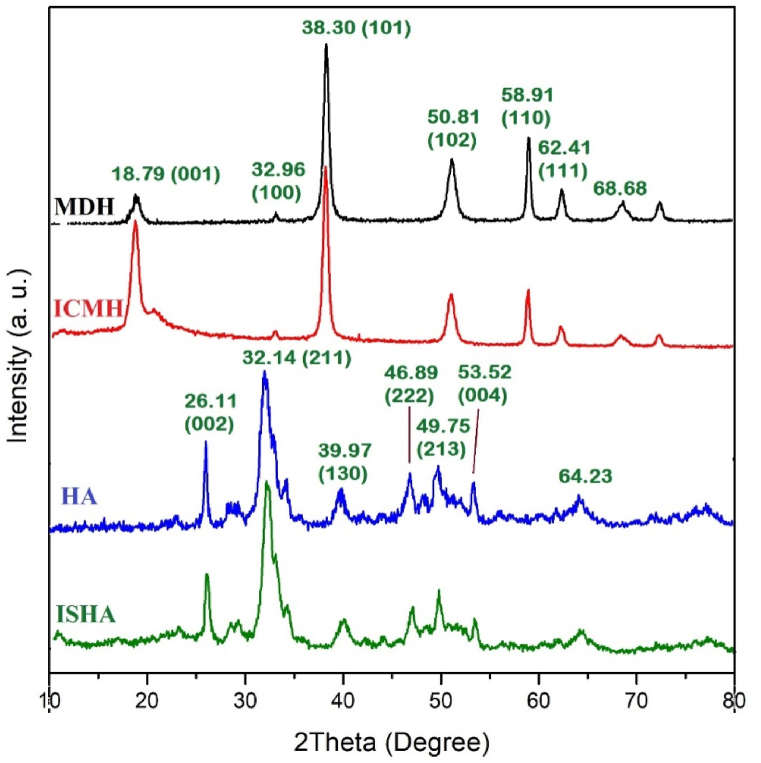
XRD patterns of MDH, ICMH, HA, and ISHA.

The XRD patterns of MDH, ICMH, HA, and ISHA are represented in Fig. 2. The XRD pattern of pure MDH exhibited prominent reflections at 2Ө = 18.79°, 32.96°, 38.30°, 50.81°, 58.91°, and 62.41° which are consistent with the (001), (100), (101), (102), (110), and (111) planes as indexed by standard JCPDS (Joint Committee on Powder Diffraction Standards) file No. [007–0239] [44]. After modification of MDH with ICCS, there were no observed shifts in the reflection positions corresponding to the MDH nanoparticles. A comparison of the two diffraction patterns suggests that surface modification has led to increased separation of the particles, likely due to the introduction of an organic structure, resulting in some broadening of the reflection peaks. Furthermore, the presence of chitosan is indicated by a broad reflection at approximately 2Ө = 20° [39]. Some reflections in the HA pattern appeared at 2θ values of 26.11°, 32.14°, 39.97°, 46.89°, 49.75°, and 53.52° are corresponded to the characteristic planes of (002), (211), (130), (222), (213), and (004) confirming the crystallin structure of HA. The prominent reflections at above angles in the XRD pattern were in good agreement with the standard diffraction data for HA, as referenced in the JCPDS database file No. [09–0432] [17]. Comparing the X-ray diffraction patterns of HA and ISHA, their XRD patterns were almost the same, indicating that the crystalline structure of HA nanoparticles remained unaffected and undamaged after surface modification with silica and imide heterocyclic structure.

**Fig. 3:**
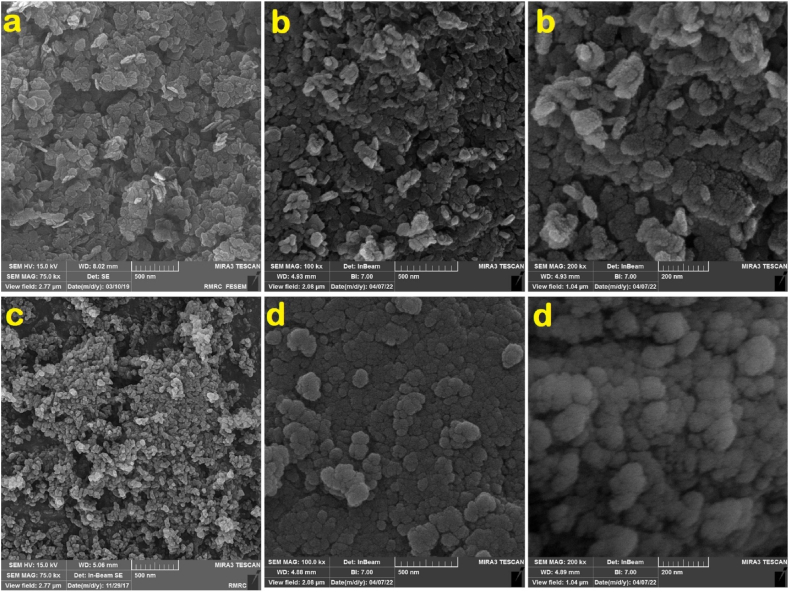
FE-SEM micrographs of a) MDH, b) ICMH, c) HA, and d) ISHA.

**Fig. 4:**
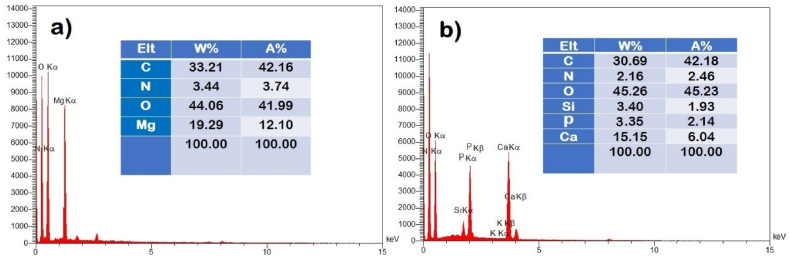
EDX spectra of a) ICMH and b) ISHA.

The FE-SEM micrographs of MDH, ICMH, HA, and ISHA are illustrated in Fig. 3. The high-resolution FE-SEM for MDH and HA exhibited a consistent shape and size, indicative of a homogeneous synthesis process. The post-modification FE-SEM images revealed an additional layer surrounding the nanoparticles, indicative of successful surface modification. Furthermore, the EDX spectra of ICMH and ISHA are shown in Fig. 4. EDX analysis of ICMH revealed distinct peaks corresponding to Mg and O, which are consistent with the expected composition of magnesium hydroxide. The EDX spectrum of ICMH exhibited additional peaks corresponding to carbon and nitrogen, indicating successful surface modification. The EDX analysis of ISHA (Fig. 4b) revealed a close correspondence with the anticipated elemental composition of the substance. Post-modification EDX analysis showed the presence of C, N, O along with Si, P, and Ca elements confirming the attachment of the organic/inorganic groups onto the HA surface.

**Fig. 5:**
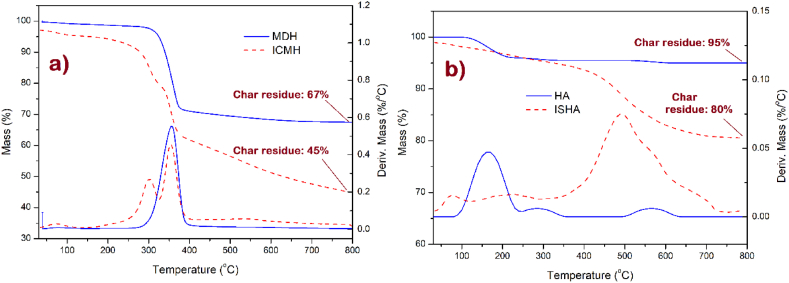
TGA/DTG thermograms of a) MDH and ICMH, b) HA and ISHA.

TGA was used to confirm the surface modification of nanoparticles with organic compounds by revealing distinct mass loss profiles at elevated temperatures. The TGA/DTG thermograms of MDH, ICMH, HA, and ISHA are shown in Fig. 5. The DTG thermograms of ICMH and ISHA exhibited an extra decomposition stage post-surface modification, validating the effective bonding of organic compounds to the mineral nanoparticle surface. Upon investigation the TGA thermograms of MDH and ICMH, it is evident that the surface of MDH has been modified with approximately 22 % of imide functionalized chitosan. MDH showed a single mass loss step around 350 °C, indicative of its transformation into MgO. Conversely, the TGA curve of ICMH displayed this primary mass loss stage alongside an additional degradation step at a lower temperature, approximately 300 °C, attributable to the degradation of the imide functionalized chitosan structure. The TGA analysis of HA revealed a dehydration stage between 100 and 150 °C, indicative of water removal from the HA structure. Likewise, the TGA curve for ISHA exhibited a dehydration phase at this temperature range, accompanied by an additional degradation step in the range of 400-600 °C. The second mass loss is attributed to the decomposition of the organic structure grafted to the nanoparticle surface. TGA results of HA and ISHA confirmed the presence of approximately 15 % organic content grafted into the surface of HA, beginning from the initial amino-silane functionalization stage.

### 3.2 Preparation and characterization of PLA/MDH/HA nanocomposites

**Scheme 2:**
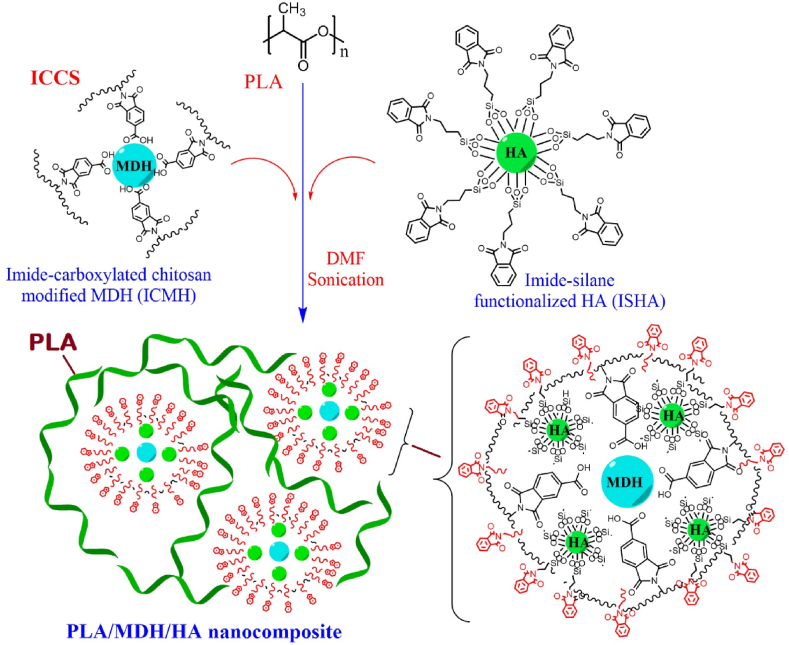
Schematic suggested preparation route of PLA/MDH/HA nanocomposites.

Within the scope of this research, MDH served as a flame retardant for PLA. To achieve this, the MDH nanoparticles underwent surface modification with imide functionalized chitosan prior to their incorporation into the PLA matrix. Although MDH effectively reduces the flammability of PLA, it also lowers its thermal resistance, as noted earlier. To counterbalance the adverse effects of MDH, herein, HA was incorporated into the PLA matrix concurrently. To enhance the compatibility, the surface of HA nanoparticles was modified using amino silane followed by the imidization. The compatibility of ICMH with PLA seems to be limited due to the polar nature of chitosan. Conversely, coating the surface of HA with an organic layer can render it hydrophobic, thereby improving its compatibility with PLA. The ISHA and ICMH mixture were anticipated to form a compatible structure with PLA. Regarding the arrangement of additive components within the polymer matrix, it can be assumed that: the HA nanoparticles orient in a way that their polar ends align towards the MDH, while the less polar ends (imide groups) orient outward. The suggested preparation rout of PLA/MDH/HA nanocomposites is shown in Scheme 2. This alignment provides the appropriate polarity to facilitate addition between the PLA chains. The structure and morphology of the PLA specimens were evaluated via FTIR, XRD, SEM, and TEM. Also, the thermal stability, mechanical properties, and combustion behavior of the specimens were studied using TGA, tensile test, and MCC.

#### 3.2.1 FTIR spectra and XRD patterns

**Fig. 6:**
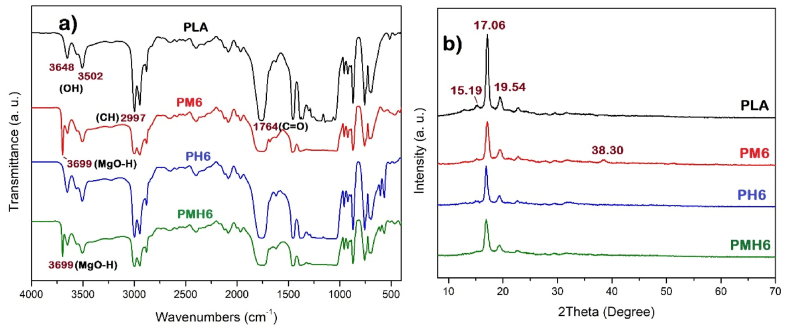
a) FTIR spectra and b) XRD patterns of the PLA samples.

The FTIR spectra of the PLA specimens are shown in Fig. 6a. The PLA spectrum displayed absorption bands that were indicative of its molecular structure. The prominent band observed at 1764 cm^−1^ corresponded to the stretching mode of the carbonyl (C=O) group, a signature feature of the ester linkage in PLA. Additionally, the absorption bands appeared near 2997 cm^−1^ can be attributed to the stretching modes of the CH groups. These bands provided the further evidence of the polymer's aliphatic nature. Furthermore, the absorption bands around 3500 cm^−1^ indicated O-H stretching vibrations, which might arise from terminal hydroxyl groups or moisture content in the sample. Because the additives are used in small quantities and their absorption bands overlapped with those of PLA, the FTIR spectra of the nanocomposites were achieved like the FTIR spectrum of pure polylactic acid. Notably, in the samples containing MDH (PM6 and PMH6), an absorption band at 3699 cm^−1^ was observed, indicating the presence of the MgO-H bond.

The XRD patterns of the PLA specimens are shown in Fig. 6b. The XRD pattern of PLA revealed distinct crystalline reflections that were characteristic of its semi-crystalline nature. The primary reflections observed at 2θ values of approximately 17.06° and 19.54° corresponded to the (110)/(200) and (203) planes of the PLA crystal structure, respectively. Amorphous regions within the PLA were observed by a broad reflection around 2θ = 10°–20°, which contrasted with the sharp reflections of the crystalline regions. Moreover, the addition of nanoparticles or other additives can influence the crystallinity of PLA, potentially leading to changes in the peak intensities or the appearance of new peaks. Such modifications in the XRD pattern can provide insights into the interactions between PLA and the incorporated materials. The XRD patterns of the nanocomposites showed that the reflections associated with the crystalline regions of PLA had reduced intensity compared to those of pure PLA. Moreover, the absence of reflections corresponding to the nano-fillers suggests that the nanoparticles were well-dispersed and uniformly distributed throughout the PLA matrix. A weak reflection at 2Ө = 38.30, in the XRD pattern of PM6, can be related to the MDH nanoparticles.

#### 3.2.2 SEM and TEM

**Fig. 7:**
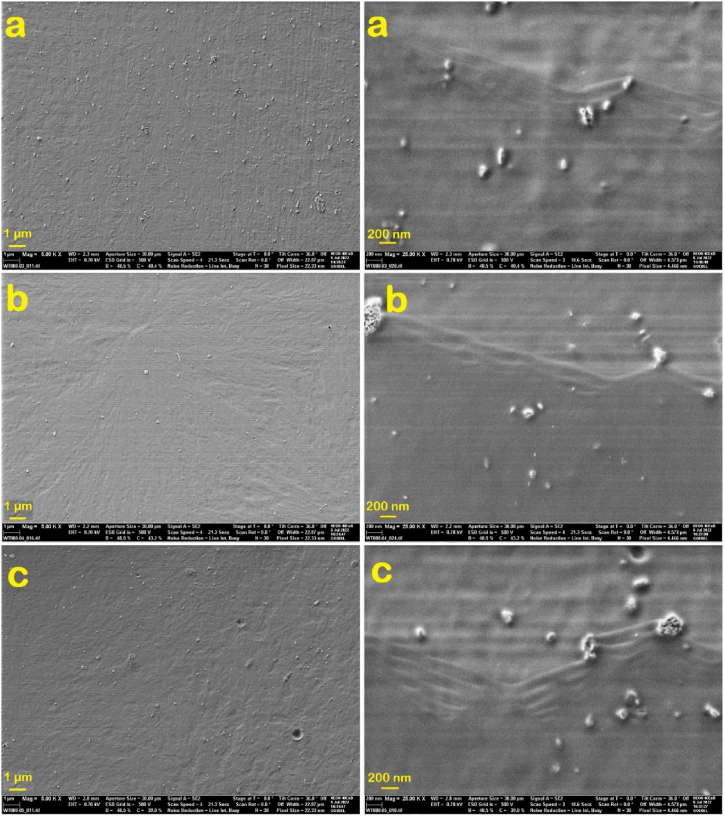
SEM micrographs of a) PM2, b) PH2, and c) PMH2.

SEM is a powerful technique used to investigate the surface morphology and dispersion quality of nanoparticles within polymer nanocomposites. The SEM micrographs of PM2, PH2, and PMH2 are shown in Fig. 7. The SEM micrographs of PM2 and PH2 nanocomposites distinctly are exhibited HA and MDH nanoparticles within the PLA matrix as bright spots. Fig. 7 illustrates that the nanoparticles are evenly dispersed throughout the polymer matrix. Nevertheless, certain regions in the PH2 nanocomposite are exhibited clusters, suggesting some degree of nanoparticle aggregation. The SEM image of PMH2 demonstrated effective interaction between the organically modified nanoparticles. Despite minor aggregation, it can be inferred that the mixed nanoparticles show enhanced compatibility within the PLA matrix compared to their separate addition. The uniform dispersion of the modified nanoparticles suggested strong interactions both among the nanoparticles and with the PLA. The surface morphology of the PLA nanocomposites, as exhibited by SEM, revealed minimal nanoparticle agglomeration, which was beneficial for maintaining the desired properties of the material.

**Fig. 8:**
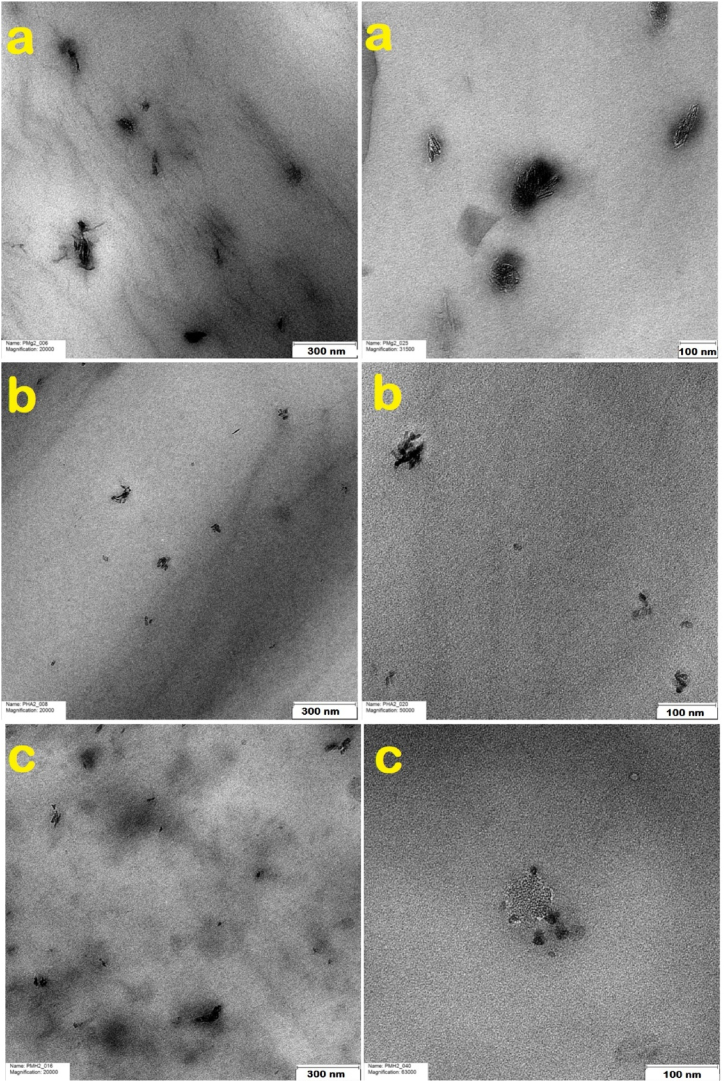
TEM images of a) PM2, b) PH2, and c) PMH2.

Analysis of TEM images revealed the nanoscale morphology of MDH and HA nanoparticles, offering valuable insights into their dispersion quality and structural integrity within the polymer nanocomposites. The TEM images of PM2, PH2, and PMH2 are shown in Fig. 8. The TEM image of the PM2 sample revealed MDH nanoparticles characterized by very small flake-like and sheet-like structures. The images demonstrated that the nanoparticles were well-dispersed within the polymer matrix. Moreover, TEM images of PH2 depicted HA nanoparticles, which exhibited a spherical shape and were significantly finer than MDH nanoparticles. These images illustrated a homogeneous structure of the PLA specimen. The TEM images of PMH2 corroborated the SEM images, exhibited the slight aggregation in some areas. However, the overarching conclusion was that the modified nanoparticles have established a structure that exhibits compatibility both among themselves and with PLA, evidenced by their effective dispersion within the polymer matrix.

#### 3.2.3 Thermal properties

**Fig. 9:**
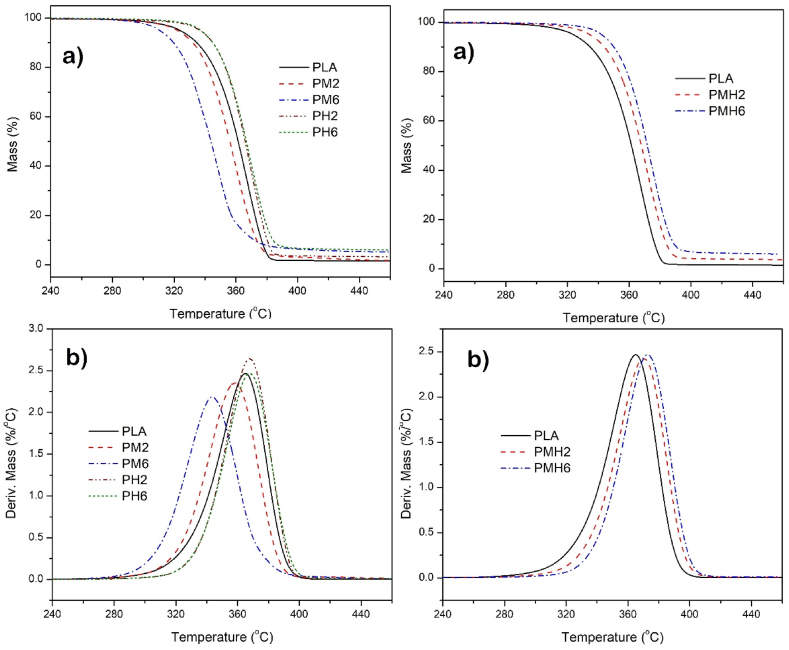
a) TGA and b) DTG thermograms of the PLA samples.

The TGA and DTG thermograms of the PLA sample films are shown in Fig. 9 and the corresponded data are listed in Table 2. The pure PLA underwent rapid and nearly complete degradation in a single stage. The temperature at 5 % and 10 % mass loss (T_5_) and the temperature at maximum decomposition (T_max_) of PLA were obtained 323 °C, 334 °C, and 364 °C, respectively. Also, the char residue for PLA was obtained almost 1 % at 700 °C. Based on the literature, the primary degradation products of PLA include oligomers, lactide, acetaldehyde, ketene, carbon monoxide, and carbon dioxide [45]. As demonstrated in Table 2, PLA samples containing ICMH exhibited reduced thermal resistance. The peak thermal decomposition temperature, along with other parameters, has also decreased. As previously mentioned, this reduction is attributed to the dehydration of MDH occurring at a temperature lower than the onset of PLA degradation. The dehydration stage of MDH and its conversion to MgO, occurring prior to the thermal decomposition of PLA, causes the composite sample to begin mass loss earlier than the pure polymer. The maximum decomposition temperature of ICMH was found to be 353 °C. This is approximately 10 °C lower than the maximum thermal decomposition temperature of PLA. In the PM2 and PM6 samples, the residual char content increased compared to pure PLA. This increase is attributed to the formation of MgO and its thermal stability as a mineral compound.

**Table 2:** Thermal properties data of the PLA samples.

Samples	Thermal properties data in N_2_ atmosphere				DSC data		
	T_5_^a^	T_10_^b^	T_max_^b^	CY^c^	T_*g*_^d^	T_m_^f^	ΔH (J.g^−1^)^g^
PLA	323	334	364	1	57	153	0.8
PM2	322	331	358	2	58	154	2
PM6	310	319	343	5	57	153	1.9
PH2	336	344	367	2.7	58	153	5.7
PH6	336	345	367	5	58	153	5.8
PMH2	334	343	369	3	57	152	3.5
PMH6	341	349	372	5	58	153	2.9

a Temperature at 5 % mass loss (°C).

b Temperature at 10 % mass loss (°C) and the main mass loss temperature (°C).

c CY: Char yield (%), Weight percentage of material left after TGA analysis at a maximum temperature of 700 °C.

d ^,f,g^ Glass transition temperature, melting temperature, and the crystallization enthalpy data, respectively, were recorded by DSC at a heating rate of 10°C/min in N_2_.

In samples containing ISHA (PH2 and PH6), the thermal resistance values increased. These results indicate that the hydroxyapatite nanoparticles, along with the effect of surface modifier on the PLA system, contribute to enhanced thermal resistance. With the increase of ISHA content in the polymer matrix, there was no significant change in the thermal resistance of the samples; however, the residual char content increased.

Simultaneous increases in ISHA and ICMH within the PLA matrix significantly enhanced the thermal resistance. The values of T_5_, T_10_, and T_max_ for the PLA sample containing 3 wt% of each additive (PMH6) were found to be 341 °C, 349 °C, and 372 °C, respectively. The thermal resistance of samples containing both additives surpassed even that of samples containing ISHA alone. These results indicate that although ICMH had an antagonism impact on thermal properties, when used individually, it exhibited a significant synergistic effect when combined with ISHA in the PLA matrix.

The DSC data from the second heating analysis are listed in Table 2. As observed in Table 2, there is no important change in the melting temperature and glass transition temperature (T_*g*_) of the samples compared to pure PLA. This indicates that the addition of nanomaterials has not significantly affected the mobility and interactions between PLA chains. However, the nanofiller loading into the PLA matrix has led to a change in the crystallinity of PLA in the nanocomposite samples, accompanied by an increase in the crystallization enthalpy.

The pure PLA showed only a slight tendency to crystallize. The crystallization enthalpy was obtained to be about 0.8 J g^−1^. The crystallization enthalpies for the PM2 and PM6 samples are slightly higher at about 2 J g^−1^. The PH2 and PH6 samples crystallized more strongly. The crystallization enthalpy was found to be almost 5.8 J g^−1^ for both samples. Additionally, the crystallization enthalpies of the PMH2 and PMH6 samples were slightly higher than the pure PLA. When nanomaterials are added as additives to the PLA matrix, it is possible for the crystallinity of the polymer base to increase. This occurs because the nanomaterials can act as nucleating agents, promoting more organized and dense crystalline structures within the PLA matrix during its solidification or crystallization process [46].

#### 3.2.4 Flame retardancy of PLA samples

**Fig. 10:**
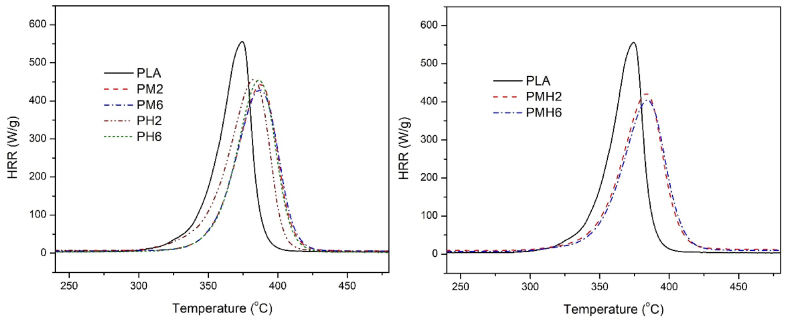
The HRR curves of the PLA samples.

Microscale combustion calorimetry (MCC) is an analytical technique widely employed to assess the flammability characteristics of polymeric materials. This method provides critical insights into the thermal degradation behavior and combustion properties of polymers, making it an invaluable tool for evaluating fire safety in various applications [47]. Several key parameters are derived from MCC analysis, including; pHRR: The maximum rate of heat release observed during the test. pHRR provides insights into the intensity of combustion at its peak. HRC: The maximum heat release rate per unit mass of the sample. HRC is a critical indicator of the material's flammability, with higher values suggesting greater fire hazard potential. THR: The cumulative heat released during the entire combustion process. THR is used to quantify the overall energy content of the material. Temperature at pHRR (T_p_): The temperature at which the peak heat release rate occurs. This parameter helps in understanding the thermal stability of the material. The HRR versus temperature curves of the as-prepared PLA samples are illustrated in Fig. 10 and the corresponded key parameters are listed in Table 3.

**Table 3:** MCC data of the PLA samples.

Samples	pHRR (W.g^−1^)^a^	HRC (J.g^−1^K^−1^)^b^	THR (KJ.g^−1^)^c^	T_p_^d^ (°C)
PLA	555.1 ± 1.2	605.2 ± 2.3	17.8 ± 0.2	374
PM2	443.3 ± 1.8	445.1 ± 3.1	16.1 ± 0.3	386
PM6	429.8 ± 2.4	440.0 ± 1.8	16.2 ± 0.1	387
PH2	456.4 ± 1.9	467.7 ± 2.8	16.9 ± 0.2	383
PH6	454.1 ± 2.6	459.5 ± 3.6	16.5 ± 0.2	385
PMH2	423.7 ± 3.1	443.1 ± 2.4	15.8 ± 0.3	383
PMH6	403.2 ± 2.7	391.4 ± 3.1	15.6 ± 0.1	387

a Heat release rate.

b Heat release capacity.

c Total heat release, and.

d The temperature at pHRR.

The pHRR, HRC and THR values of the pure PLA were found to be 555.1 W g^−1^, 605.2 J g^−1^K^−1^, and 17.8 kJ g^−1^, respectively. As shown in Table 3, the addition of ICMH and ISHA individually to PLA resulted in a reduction of all three aforementioned parameters. Notably, the samples containing MDH exhibited lower values for the parameters pHRR, THR, and HRC.

The flame retardancy mechanism of MDH primarily involves its endothermic decomposition process during heating, which occurs around 330 °C. MDH releases water molecules and converts into MgO, absorbing a significant amount of heat and reducing the temperature of the polymer matrix. This endothermic reaction dilutes flammable gases and inhibits the combustion chain reaction, effectively delaying the ignition and flame propagation of the polymer. Additionally, the formation of a protective layer of MgO on the polymer surface further enhances its flame-retardant properties by shielding the underlying material from heat and oxygen exposure [48]. These mechanisms collectively contribute to the improved fire safety performance observed in polymer composites containing MDH. On the other hand, Hydroxyapatite exhibits significant flame-retardant properties by promoting the formation of a stable char layer during combustion. This char layer acts as a barrier, reducing heat transfer and hindering the spread of flames, thereby enhancing the fire resistance of the polymer matrix [18,22].

The PLA containing 3 wt% of ICMH and 3 wt% of ISHA (PMH6) exhibited the lowest content for the key parameters including pHRR, HRC, and THR. These results demonstrate that the simultaneous presence of MDH and HA, along with organic compounds such as imidized chitosan and amino propyl silane, synergistically reduced the flammability of PLA. Based on the results, it appears that MDH had a greater effect in reducing the parameters obtained from MCC analysis. The simultaneous presence of MDH and HA not only reduced the flammability of PLA but also increased its thermal resistance. This observation can be inferred from both the TGA curves and the temperature values at PHRR of the samples. The T_p_ of the pure PLA was obtained to be 374 °C, while for PMH6 was found to be 387 °C. Generally, the T_p_ values of the samples were higher than the T_max_ values obtained from TGA. At the main mass loss temperature indicated by TGA, the polymer structure decomposed through heat absorption, whereas at T_p_, the absorbed heat from the polymeric sample was released into the gas phase [17]. The difference in heating rates (10 °C.min-1 for TGA vs. 60 °C.min^−1^ for MCC) could explain these findings [49].

The flame retardancy of PLA nanocomposite samples is further assessed through the LOI and UL-94 tests. The outcomes of these tests are summarized in Table 4, and the images corresponding to the UL-94 test for pure PLA, PM6, and PMH6 are displayed in Fig. S1. The LOI value of neat PLA was found to be 19.2 %, while the LOI values for PM6, PMH2, and PMH6 were 24.4 %, 22.7 %, and 26.8 %, respectively.

**Table 4:** LOI and UL94 test results of the PLA samples.

Sample	LOI^a^ (%)	UL-94 Rating	Dripping
PLA	19.2	No rating	Yes
PM2	-b	No rating	Yes
PM6	24.4	V-2	Yes
PH2	–	No rating	Yes
PH6	20.3	No rating	Yes
PMH2	22.7	No rating	Yes
PMH6	26.8	V-1	No

a Limiting oxygen index.

b Not measured.

Despite the PMH2 sample exhibiting a higher LOI value compared to pure PLA, it did not achieve any UL-94 rating. In the case of PM6, the suppression of heavy dripping led to a V-2 rating. PMH6, which showed the highest LOI value, was extinguished in less than 30 s during the UL-94 test without any dripping. The findings suggest that MDH had a more pronounced effect on enhancing flame retardancy, while the modified HA demonstrated a synergistic interaction with modified MDH, further reducing the flammability of PLA.

#### 3.2.5 Tensile test

**Fig. 11:**
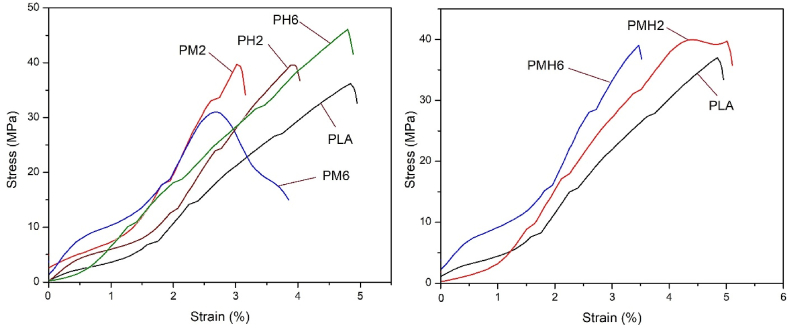
Stress-strain curves of the PLA samples.

Tensile testing is a fundamental test, which used to determine how materials will react under tension, measuring their strength and ductility. This test involves stretching a material until it breaks to assess its mechanical properties, such as ultimate tensile strength and elongation. The tensile curves of the PLA specimens are depicted in Fig. 11 and the related key parameters amounts are listed in Table 5.

**Table 5:** Tensile properties data of the PLA samples.

Samples	T_s_ (MPa)^a^	EB (%)^b^	YM (GPa)^c^
PLA	36.04 ± 0.63	4.84 ± 0.41	2.61 ± 0.21
PM2	39.54 ± 0.56	3.01 ± 0.31	3.03 ± 0.32
PM6	30.9 ± 0.81	3.71 ± 0.29	2.99 ± 0.27
PH2	39.44 ± 0.89	3.88 ± 0.43	3.01 ± 0.24
PH6	46.23 ± 0.78	4.79 ± 0.24	2.71 ± 0.19
PMH2	39.0 ± 0.85	4.98 ± 0.39	2.72 ± 0.25
PMH6	38.58 ± 0.67	3.41 ± 0.27	3.01 ± 0.21

a Tensile strength.

b Elongation at break.

c Young's modulus.

According to the results presented in Table 5, the tensile strength enhances with the addition of ICMH up to 2 % by mass; however, when the loading reaches 6 %, there is a noticeable reduction in tensile strength. This phenomenon can be explained by the influence of MDH nanoparticles and the potential aggregation that happens at higher loading. Notably, incorporating ISHA into the PLA matrix has led to an enhancement in tensile strength. Incorporating 2 % by mass of ISHA into the PLA matrix enhanced the tensile strength from 36.04 MPa to 39.44 MPa, while incorporating 6 % increased the tensile strength to 46.23 MPa. These findings suggest that the ISHA filler demonstrates superior compatibility with the PLA matrix compared to ICMH and probably experiences less aggregation. The strong forces between the PLA chains and the functional groups in ISHA have contributed to the enhanced tensile strength of the nanocomposite. The impact of both fillers is evident in the PMH2 and PMH6 samples. Incorporating up to 2 % by mass of the filler mixture increased the tensile strength from 36.04 MPa to 39.0 MPa, whereas loading 6 % led to a reduction in tensile strength. This decrease can be attributed, based on previous results, to the presence of ICMH.

The results indicated a reasonable relationship between filler loading and both Young's modulus (YM) and elongation at break (EB). In sample PMH6, the YM value increased from 2.61 GPa to 3.01 GP compared to pure PLA, which is confirmed by the decrease in EB from 4.84 % to 3.41 %. The loading fillers into PLA, either individually or in combination, has resulted in a substantial increase in YM due to various forces in functional groups in the fillers and PLA chains. These intermolecular interactions have decreased the flexibility of the resulting nanocomposites. The increase in the YM values, accompanied by reduced flexibility, has resulted in brittleness of the samples. Therefore, loading fillers has led to decreased values of EB.

## 4 Conclusions

The surface of hydroxyapatite (HA) and magnesium dihydroxide (MDH) nanoparticles were organically modified with silane-imide structure and imide-carboxylated functionalized chitosan, respectively, and used for the preparation of the PLA nanocomposites. The impact of simultaneously and individually of imide-carboxylated chitosan modified MDH (ICMH) and imide-silane functionalized HA (ISHA) were investigated on the thermal and mechanical properties as well as the flammability of the PLA matrix. The XRD patterns, SEM micrographs and TEM images of the as-prepared PLA nanocomposites indicated that the surface modified nanoparticles had a desirable dispersion in the PLA matrix. The TGA results showed due to the proximity of the mass loss temperature of MDH to the PLA degradation temperature, the ICMH filler did not have a significant effect on thermal properties. However, when ICMH was combined with ISHA as PLA additives, it notably enhanced thermal resistance. The T_10_ and T_max_ values of the PLA sample with 3 wt% of each filler increased from 334 °C to 349 °C and from 364 °C to 372 °C, respectively, as compared to pure PLA. The impact of modified MDH on the flammability of PLA samples was significant. According to MCC results, modified MDH had a more pronounced impact on the flammability of PLA samples compared to modified HA. The pHRR and HRC values for the PMH6 sample decreased from 555.1 W g^−1^ to 403.2 W g^−1^ and from 605.2 J g^−1^K^−1^ to 391.4 J g^−1^K^−1^, respectively, as compared to pure PLA. The LOI value PLA increased from 19.2 % to 26.8 % and UL-94 V-1 rating with increasing modified MDH and HA contents up to 6 wt%. Specifically, MDH acts as a supplementary additive to diminish flammability, while HA offsets the decrease in thermal resistance introduced by MDH. From tensile test exhibited an improvement mechanical property by nano structure loading into the PLA matrix. In conclusion, the production of PLA nanocomposites with hydroxyapatite and magnesium hydroxide nanoparticles represents a promising avenue for creating multifunctional materials with potential applications across various industries.

## CRediT authorship contribution statement

**Mohsen Hajibeygi:** Writing – review & editing, Writing – original draft, Supervision, Project administration, Investigation, Formal analysis, Conceptualization. **Fatemeh Darvishi:** Writing – original draft, Investigation, Formal analysis.

## Data availability

Data will be made available on request.

## Declaration of competing interest

The authors declare that they have no known competing financial interests or personal relationships that could have appeared to influence the work reported in this paper.
