# Supplementary figures and images for: Inclusion of modified nano-magnesium hydroxide as an adjuvant flame retardant in the development of PLA/hydroxyapatite nanocomposites
Source: Heliyon. 2024 Oct 18;10(20):e39529. doi: 10.1016/j.heliyon.2024.e39529 (PMC11533608; doi:10.1016/j.heliyon.2024.e39529)

Supplementary materials


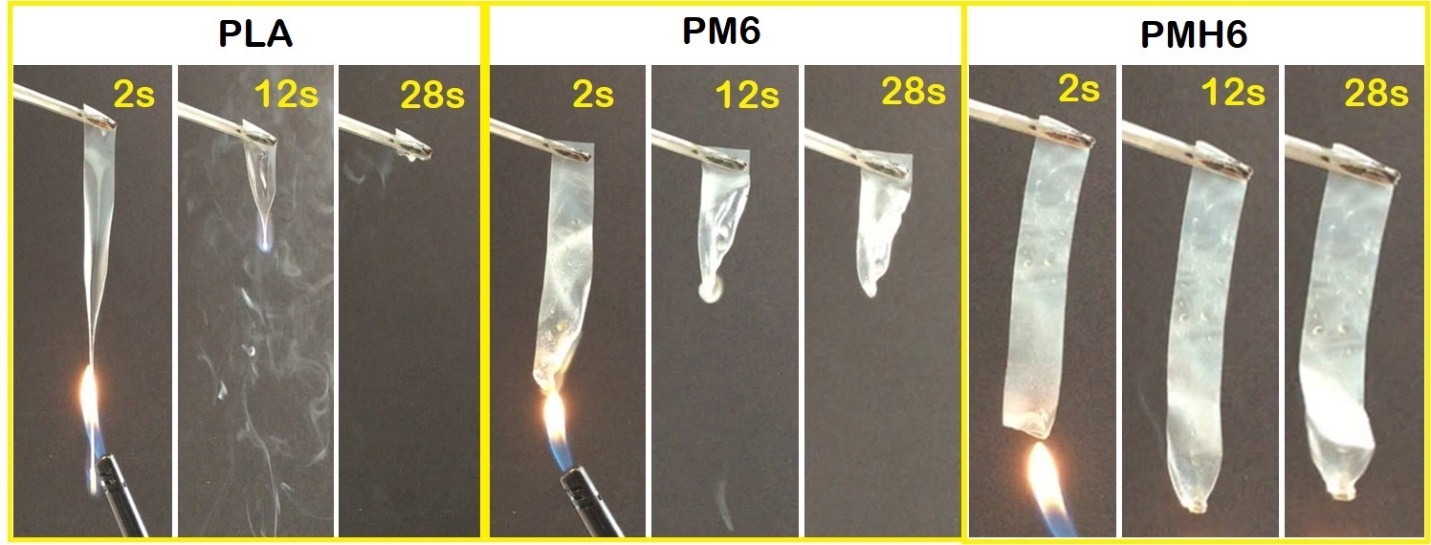


**Figure S1.** The UL94 test images of PLA, PMH2, and PMH6

Supplement: Multimedia component 1 [file mmc1.docx]
